# Blood metabolomic and postpartum depression: a mendelian randomization study

**DOI:** 10.1186/s12884-024-06628-3

**Published:** 2024-06-14

**Authors:** Keng Ling, Minping Hong, Liqin Jin, Jianguo Wang

**Affiliations:** 1 Jiaxing Women and Children’s Hospital, Wenzhou Medical University, Jiaxing, China; 2 Jiaxing Hospital of Traditional Chinese Medical, Jiaxing, China; 3 Central Laboratory, Jiaxing Maternity and Child Health Care Hospital, Affiliated Women and Children Hospital, Jiaxing University, Jiaxing, 314000 China

**Keywords:** Postpartum depression, Metabolomics, Mendelian randomization

## Abstract

**Background:**

Postpartum depression is a complex mental health condition that often occurs after childbirth and is characterized by persistent sadness, anxiety, and fatigue. Recent research suggests a metabolic component to the disorder. This study aims to investigate the causal relationship between blood metabolites and postpartum depression using mendelian randomization (MR).

**Methods:**

This study used a bi-directional MR framework to investigate the causal relationship between 1,400 metabolic biomarkers and postpartum depression. We used two specific genome-wide association studies datasets: one with single nucleotide polymorphisms data from mothers diagnosed with postpartum depression and another with blood metabolite data, both of which focused on people of European ancestry. Genetic variants were chosen as instrumental variables from both datasets using strict criteria to improve the robustness of the MR analysis. The combination of these datasets enabled a thorough examination of genetic influences on metabolic profiles associated with postpartum depression. Statistical analyses were conducted using techniques such as inverse variance weighting, weighted median, and model-based estimation, which enabled rigorous causal inference from the observed associations. postpartum depression was defined using endpoint definitions approved by the FinnGen study’s clinical expert groups, which included leading experts in their respective medical fields.

**Results:**

The MR analysis identified seven metabolites that could be linked to postpartum depression. Out of these, one metabolite was found to be protective, while six were associated with an increased risk of developing the condition. The results were consistent across multiple MR methods, indicating a significant correlation.

**Conclusions:**

This study emphasizes the potential of metabolomics for understanding postpartum depression. The discovery of specific metabolites associated with the condition sheds new insights on its pathophysiology and opens up possibilities for future research into targeted treatment strategies.

**Supplementary Information:**

The online version contains supplementary material available at 10.1186/s12884-024-06628-3.

## Introduction

Postpartum depression is a serious mental health condition that affects new mothers [1, 2]. It is characterized by persistent feelings of sadness, anxiety, and fatigue, making it difficult for the affected women to perform daily care activities for themselves or their newborns [3]. Other symptoms may include altered sleeping and eating habits, extreme irritability, and feeling of worthlessness or guilt [4]. This condition has an impact not only on the mother’s health but also on the baby’s bonding and development [5]. Early detection and treatment are critical to improve outcomes for both mother and child [6, 7]. The cause of postpartum depression is not fully understood, but recent research has begun to look into the role of metabolic changes in its development. Specifically, changes in blood metabolites have been linked to the onset and progression of postpartum depression, implying a potential metabolic component to the disorder [8–12]. However, the causality of this relationship remains uncertain.

Blood metabolomics, which involves a thorough examination of metabolites in biological systems, has emerged as a valuable methodology for understanding the complex interplay between genetic factors, environmental influences, and disease processes [13–15]. This analytical approach, which examines metabolite configurations, provides important insights into the metabolic anomalies associated with postpartum depression. Nonetheless, the field’s reliance on observational studies frequently presents difficulties due to confounding factors and issues of reverse causation.

The use of mendelian randomization (MR) in this context provides a distinct methodological advantage. MR uses genetic variants as instrumental variables (IVs), providing a novel way to decipher causal relationships between various exposures and outcomes while avoiding the inherent confounders and biases found in observational studies. Our study uses the MR paradigm to investigate the potential causal relationship between metabolomic profiles and postpartum depression. Consequently, this study stands out as a pioneering effort in this emerging research arena, with the potential to significantly enrich our understanding of postpartum depression and its broader implications.

## Materials and methods

### Study Methodology

In this study, we used a bi-directional two-sample MR approach to investigate the hypothesized causal relationship between 1,091 blood-derived metabolites, 309 metabolite ratios, and postpartum depression [16]. The MR paradigm uses genetic variants that are harnessed as surrogate markers for potential risk factors. To derive credible causal inferences using IVs, three cardinal tenets must be followed:

1. There is a direct relationship between genetic variation and exposure.
2. There is no link between these genetic variants and confounders that could influence the exposure-outcome interaction.
3. Exclusiveness in the impact of genetic variation-driven exposure on outcome.

### Data sources for postpartum depression through genome-wide association studies (GWAS)

Summary statistics pertinent to postpartum depression were obtained from the GWAS database (https://gwas.mrcieu.ac.uk/). For postpartum depression, the sample consisted of 67,205 mothers with complete postpartum follow-up records, including 7,604 cases and 59,601 controls, totaling approximately 16,376,275 single nucleotide polymorphisms (SNPs). The study’s population was composed of people of European descent. Postpartum depression was defined using endpoint definitions approved by the FinnGen study’s clinical expert groups, which included leading experts in their respective medical fields [17]. The FinnGen study established a strong framework for defining medical conditions, ensuring consistency and reliability in our diagnostic criteria [18, 19].

### Sources of GWAS data for 1,091 blood metabolites and 309 metabolite ratios

The GWAS database, available at https://gwas.mrcieu.ac.uk/, served as a repository for summary statistics across a wide range of conditions. The GWAS summary datasets for 1,400 metabolites were extracted from the seminal study conducted by Chen et al [16], which represented the most extensive exploration to date of genetic influences on human serum metabolism. The exhaustive list of these 1,400 metabolites is provided in Supplementary Table S1. The demographic focus of this study was on people with European ancestry.

### IVs Selection

Consistent with the current scientific literature, we set a significance threshold of IVs related to each trait at 1 × 10^− 5^ [20, 21]. This process was made easier by using the R package “TwoSampleMR” [22], which helped refine the selection of SNPs.

- Clumping for Linkage Disequilibrium.

To address linkage disequilibrium (LD) between SNPs, we set a threshold of r^2 at 0.001 and specified a clumping proximity of 10,000 kb. LD measures the non-random association of alleles at different loci in a population, with an r^2 value of 1 indicating complete LD and a value of 0 indicating no LD.

b) Assessment of Instrument Strength.

To mitigate bias stemming from weak instruments, we calculated the ${R}^{2}$ and F statistics for each SNP. The ${R}^{2}$ statistic quantifies the proportion of exposure variance explained by the IV, providing insight into the strength of the instrument. Conversely, the $F$ statistic evaluates the overall instrument strength, incorporating both the ${R}^{2}$ value and the sample size of the exposure group. SNPs with an F-statistic below 10 were excluded from subsequent analyses to minimize the risk of weak instrument bias, which can lead to inflated type I error rates and unreliable estimates.To calculate ${R}^{2}$ and $F$ statistics, follow the steps below:

$$\begin{array}{l}{R^2} = \\\frac{{2\beta _{exposure}^2ea{f_{exposure}}\left( {1 - ea{f_{exposure}}} \right)}}{{2\beta _{exposure}^2ea{f_{exposure}}\left( {1 - ea{f_{exposure}}} \right) + 2se_{exposure}^2samplesiz{e_{exposure}}ea{f_{exposure}}\left( {1 - ea{f_{exposure}}} \right)}}\end{array}$$

$$F = \frac{{{R^2}\left( {samplesiz{e_{exposure}} - 2} \right)}}{{1 - {R^2}}}$$

In these formulae:

- *β*_exposure_ represents the beta coefficient of exposure,
- *eaf*_*exposure*_ is the effect of allele frequency of exposure.
- *se*_*exposure*_ represents the standard error of exposure.
- *samplesize*_*exposure*_ indicates the sample size of the exposure group.

We excluded SNPs with an F-statistic of less than 10 from our analysis to avoid weak instrument bias, which can inflate type I error rates and produce unreliable estimates [23, 24].

### Statistical approach

Analyses were conducted using the R programming environment (version 4.3.1). The investigation into the causal relationship between 1,400 metabolites and postpartum depression was executed using methodologies such as inverse variance weighting (IVW) [25], weighted median [26], and mode-based techniques [27], primarily through the TwoSampleMR package. Cochran’s Q statistic was used to investigate heterogeneity among the IVs. In cases of significant heterogeneity, the random-effects IVW model replaced the fixed-effects model. The MR-Egger method was used to address potential horizontal pleiotropy, with the intercept serving as an indicator of its existence [28]. Furthermore, the MR-PRESSO approach was used to refine the analysis by identifying and eliminating outliers that could be attributed to pleiotropy. Funnel plots were used to ensure the consistency and reliability of the findings. To summarize, the screening of blood metabolites for potential causal impact on postpartum depression was stringently conducted based on multiple criteria: (1) a significant *p*-value in the primary analysis (IVW derived *p* < 0.05 and FDR < 0.5), (2) consistent direction and magnitude across the five MR methods, and (3) absence of heterogeneity or horizontal pleiotropy in the MR results.

## Results

Probing the Impact of Metabolites on Postpartum Depression.

Based on the predefined criteria for selecting IVs, a comprehensive set of 19,541 SNPs were used as IVs in this study. Detailed information about these chosen SNPs and their characteristics are contained in Supplemental Table S2.

**Fig. 1:**
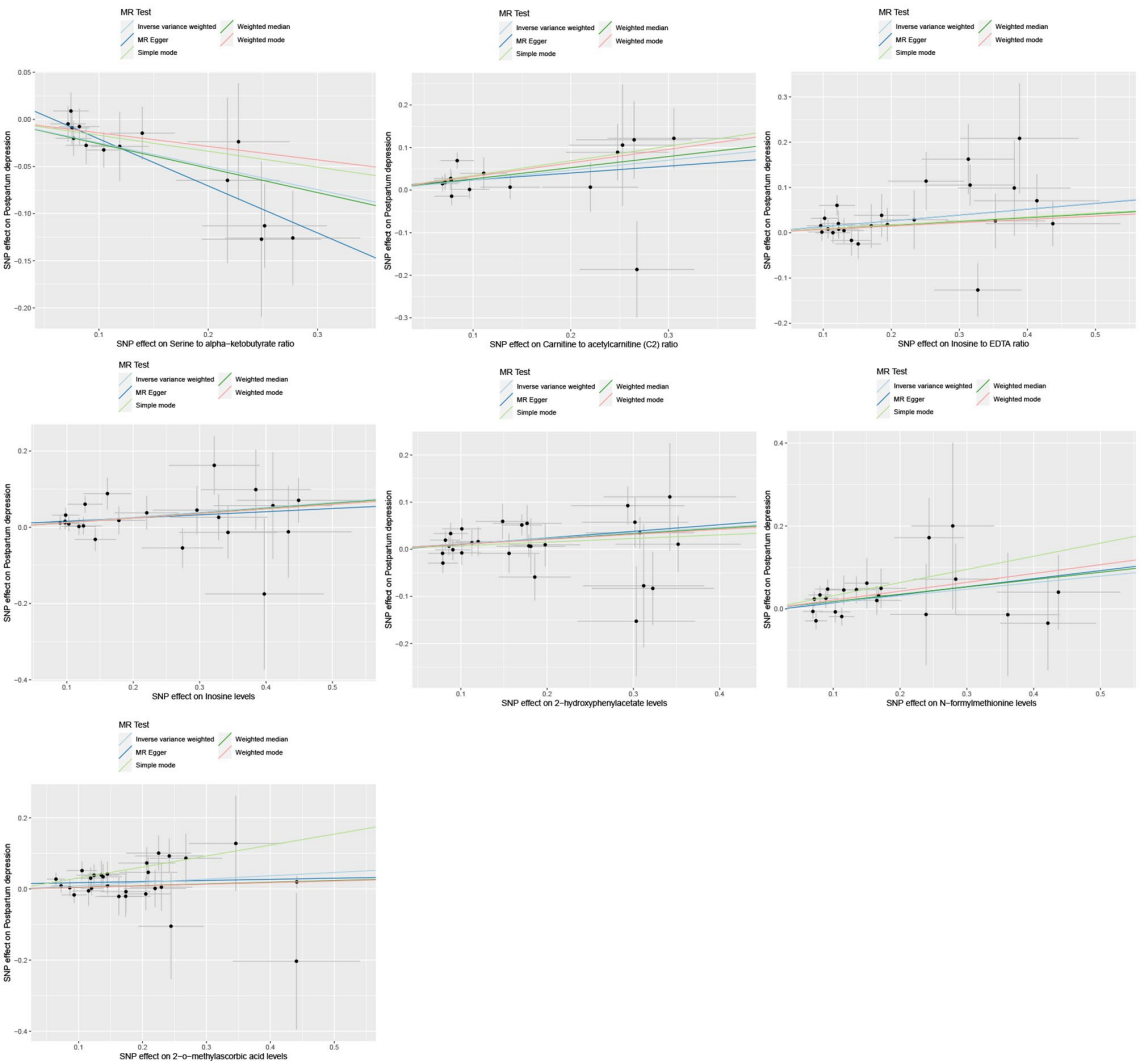
Scatter plots for the causal association between metabolites and postpartum depression

**Fig. 2:**
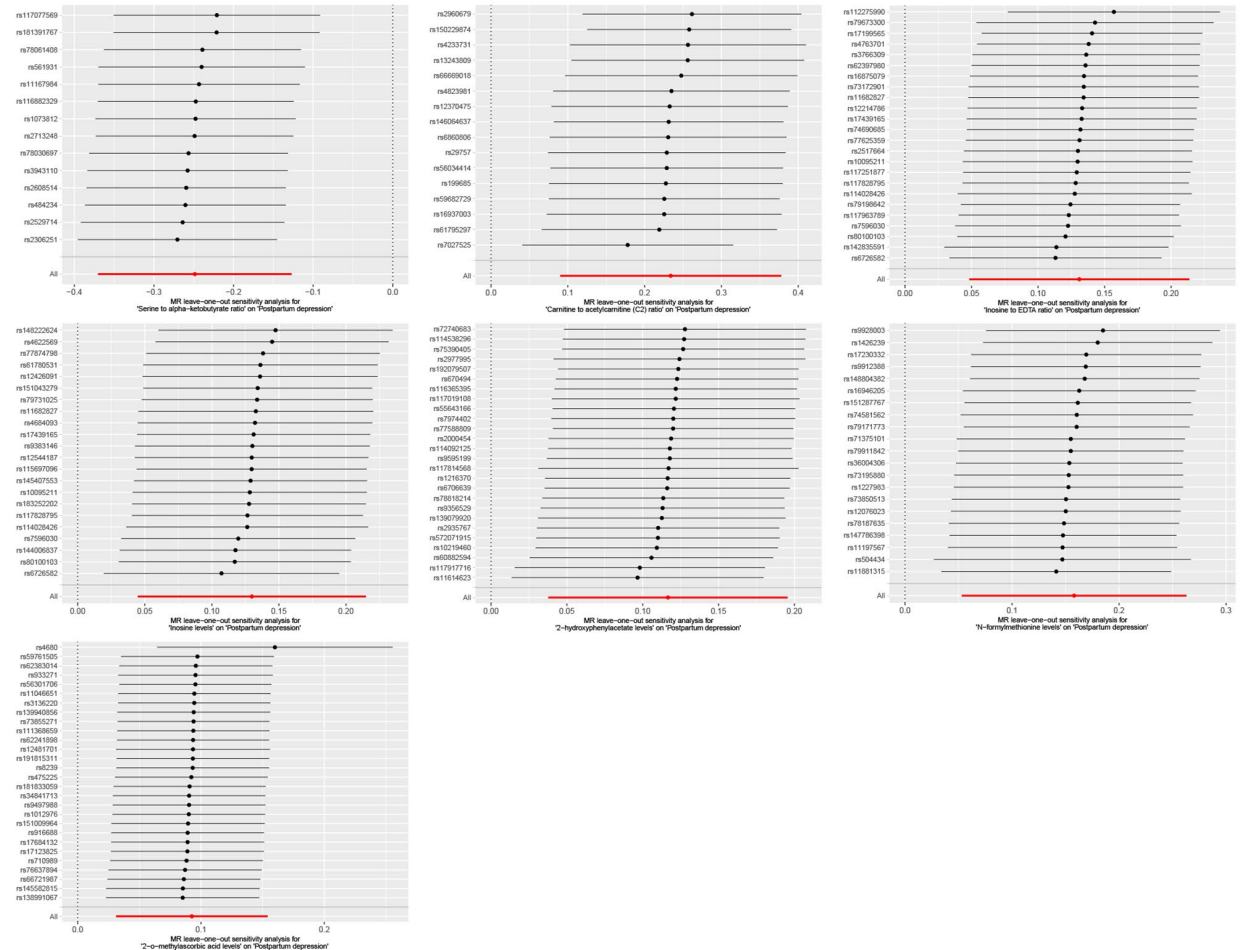
Leave-one-out plots for the causal association between metabolites and postpartum depression. The consistency of results remains robust even after excluding individual genetic variants in each analysis, indicating a high level of reliability and stability in our findings

An MR study identified seven metabolites. One of these metabolites showed protective effects against postpartum depression, while seven metabolites were found to have potential pathogenic roles (Figs. 1 and 2). The full results and detailed data of the MR analysis are contained in Supplemental Table S3.

The IVW analysis for these metabolites yielded an aggregated score of:

2-o-methylascorbic acid levels (OR 1.10, *p* = 0.003188963),

N-formylmethionine levels (OR 1.17, *p* = 0.003186648),

2-hydroxyphenylacetate levels (OR 1.12, *p* = 0.003777128),

Inosine levels (OR 1.14, *p* = 0.002786266),

Inosine to EDTA ratio (OR 1.14, *p* = 0.001912067),

Carnitine to acetylcarnitine (C2) ratio (OR 1.26, *p* = 0.001447855).

Serine to alpha-ketobutyrate ratio (OR 0.78, *p* = 6.20E-05).

The Cochran’s IVW Q test, as detailed in Supplemental Table S4, revealed no significant heterogeneity in the IVs (*p* > 0.05). Furthermore, the MR-Egger regression intercept analysis, as shown in Supplemental Table S5, found no significant directional horizontal pleiotropy (*p* > 0.05). Furthermore, the MR-PRESSO global test (results in Supplemental Table S6) found no significant outliers, indicating a negligible presence of horizontal pleiotropy in the relationship between metabolites and postpartum depression (*p* > 0.05).

**Fig. 3:**
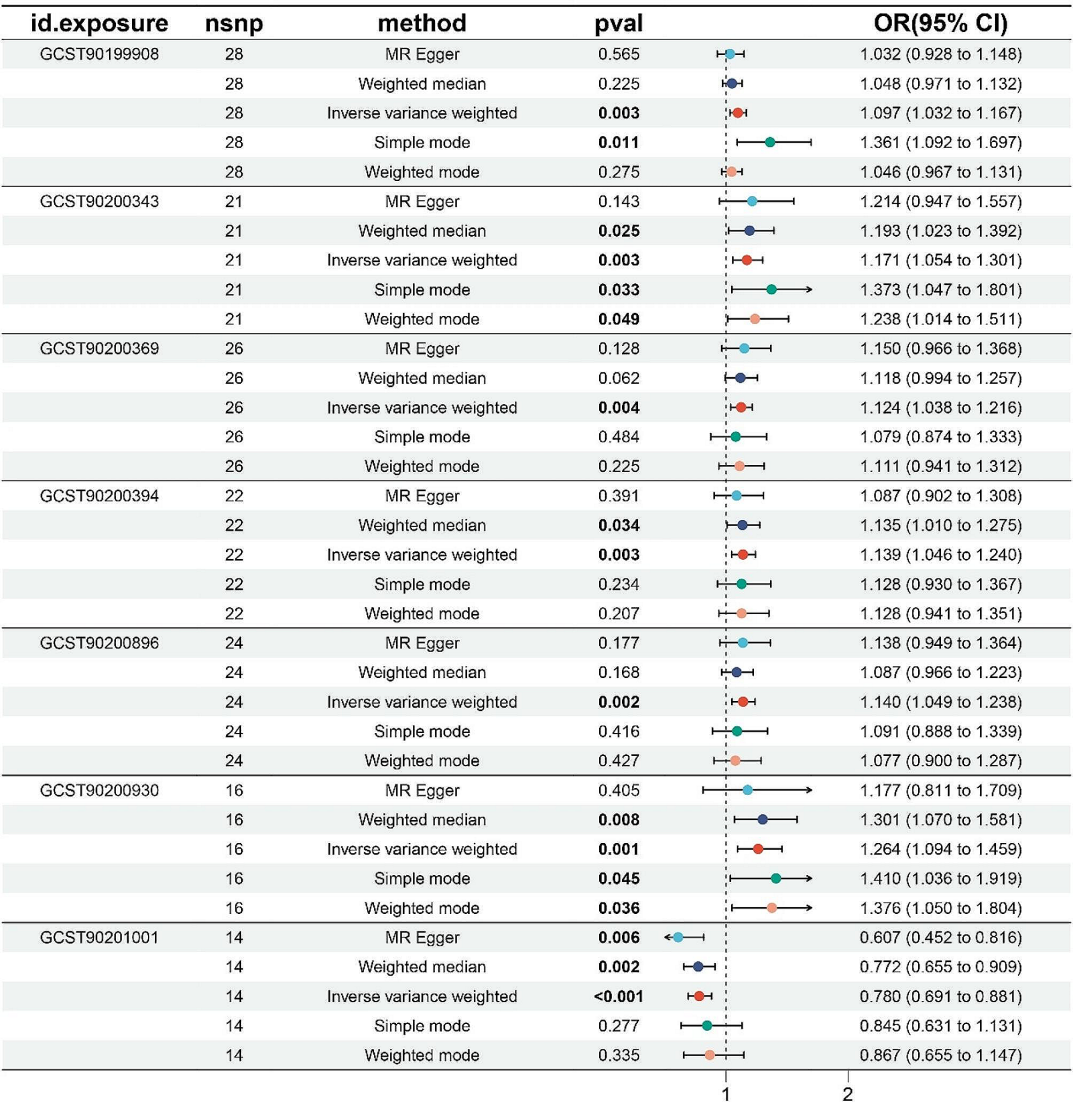
Forest plots showed the causal associations between metabolites on postpartum depression. IVW: inverse variance weighting; CI: confidence interval; GCST90201001:Serine to alpha-ketobutyrate ratio; GCST90200930:Carnitine to acetylcarnitine (C2) ratio; GCST90200896:Inosine to EDTA ratio; GCST90200394:Inosine levels; GCST90200369:2-hydroxyphenylacetate levels; GCST90200343:N-formylmethionine levels; GCST90199908:2-o-methylascorbic acid levels; nsnp: the number of SNPs involved in each MR study

Notably, the IVW analysis produced significant estimates (*p* < 0.05), and the direction and magnitude of the estimates were consistent across four other analytical methods. (Fig. 3).

## Discussion

In this study, we used a comprehensive approach that included GWAS data to investigate the causal relationships of 1,091 blood metabolites and 309 metabolite ratios with postpartum depression using a strong MR framework. Our study identified specific metabolites that were linked to an increased risk of postpartum depression. Notably, the ratio of Serine to alpha-ketobutyrate was found to be inversely correlated with the risk of postpartum depression. Conversely, a genetic predisposition to higher levels of 2-o-methylascorbic acid, N-formylmethionine, 2-hydroxyphenylacetate, and Inosine, as well as the ratios of Inosine to EDTA and Carnitine to acetylcarnitine (C2), has been linked to an increased risk of postpartum depression. These novel findings contribute significantly to a better understanding of postpartum depression’s pathophysiology, emphasizing the importance of metabolic pathways in disease risk.

In our review of the literature, we discovered that certain metabolites, such as Inosine and Serine, are mentioned concerning postpartum depression, albeit not always as the primary focus of the study. For example, while Inosine is a metabolite in the purine metabolic pathway that is involved in various psychiatric disorders, its direct link to Postpartum depression is not well documented [29]. However, given the broader implications of purine metabolism in psychiatric conditions, Inosine’s role could be interpreted as potentially relevant to postpartum depression, warranting further investigation [30–34]. The purine pathway, which is frequently altered in metabolic conditions such as hyperuricemia and can be influenced by pregnancy-related conditions such as preeclampsia, suggests a mechanism by which obstetric complications may influence the risk of postpartum depression via metabolic disruptions [35, 36].

Similarly, while the Serine to alpha-ketobutyrate ratio is not directly linked to postpartum depression in the available literature, Serine metabolism is recognized for its involvement in several psychiatric disorders, suggesting an avenue for further research into its specific role in postpartum depression [37–40]. Gestational diabetes, for example, can alter Serine and other amino acid levels, implying that metabolic diseases during pregnancy may predispose women to postpartum depression by disrupting key metabolic pathways involved in mood regulation [41, 42].

Conversely, our searches yielded no direct evidence linking metabolites such as 2-o-methylascorbic acid, N-formylmethionine, 2-hydroxyphenylacetate, the Inosine to EDTA ratio, or the Carnitine to acetylcarnitine (C2) ratio with postpartum depression. This does not rule out their involvement, but it does highlight the need for more research into the potential links between these metabolites and postpartum depression. The lack of direct associations in the literature suggests that these metabolites’ roles in Postpartum depression are unknown, and additional research could greatly contribute to our understanding of postpartum depression’s pathophysiology. This could include looking into how these metabolites interact with other metabolic pathways, as well as their potential role in the onset or progression of postpartum depression.

Our study stood out for its use of comprehensive blood metabolite GWAS data, which is uncommon in previous postpartum depression research. Furthermore, the MR approach we used yielded strong evidence for investigating the causal relationship between metabolites and postpartum depression. The use of this method not only increased our understanding of the disease but also provided new avenues for future treatment methods.

However, our study does have limitations. Firstly, due to the small number and diversity of samples, our findings may require validation in a larger population. Furthermore, while the MR approach can reduce confounding and reverse causation, it still requires strong genetic IVs. Therefore, future studies should include larger sample sizes and more genetic variants to improve the reliability and universality of our findings.

In conclusion, our findings provide new insights into the metabolic mechanisms underlying postpartum depression and may pave the way for future treatment strategies. Future research should validate the role of these metabolites in the onset of postpartum depression and investigate their potential therapeutic applications. Furthermore, research must be expanded to include different populations and a broader range of metabolites to fully comprehend the biological basis of this complicated disease.

## Conclusions

Our study, which used comprehensive blood metabolite GWAS data and mendelian randomization, found significant associations between specific metabolites and the risk of postpartum depression. We discovered metabolites associated with both increased and decreased risk, providing new insights into the disease’s metabolic basis.

### Electronic supplementary material

Below is the link to the electronic supplementary material.

Supplementary Material 1

Supplementary Material 2

Supplementary Material 3

## Data Availability

The datasets analyzed during the current study are available in the GWAS repository. The specific datasets used are: Postpartum depression: finn-b-O15_POSTPART_DEPR Blood Metabolomic: Supplemental Table S1
