# Supplementary figures and images for: Blood metabolomic and postpartum depression: a mendelian randomization study
Source: BMC Pregnancy Childbirth. 2024 Jun 14;24:429. doi: 10.1186/s12884-024-06628-3 (PMC11177545; doi:10.1186/s12884-024-06628-3)

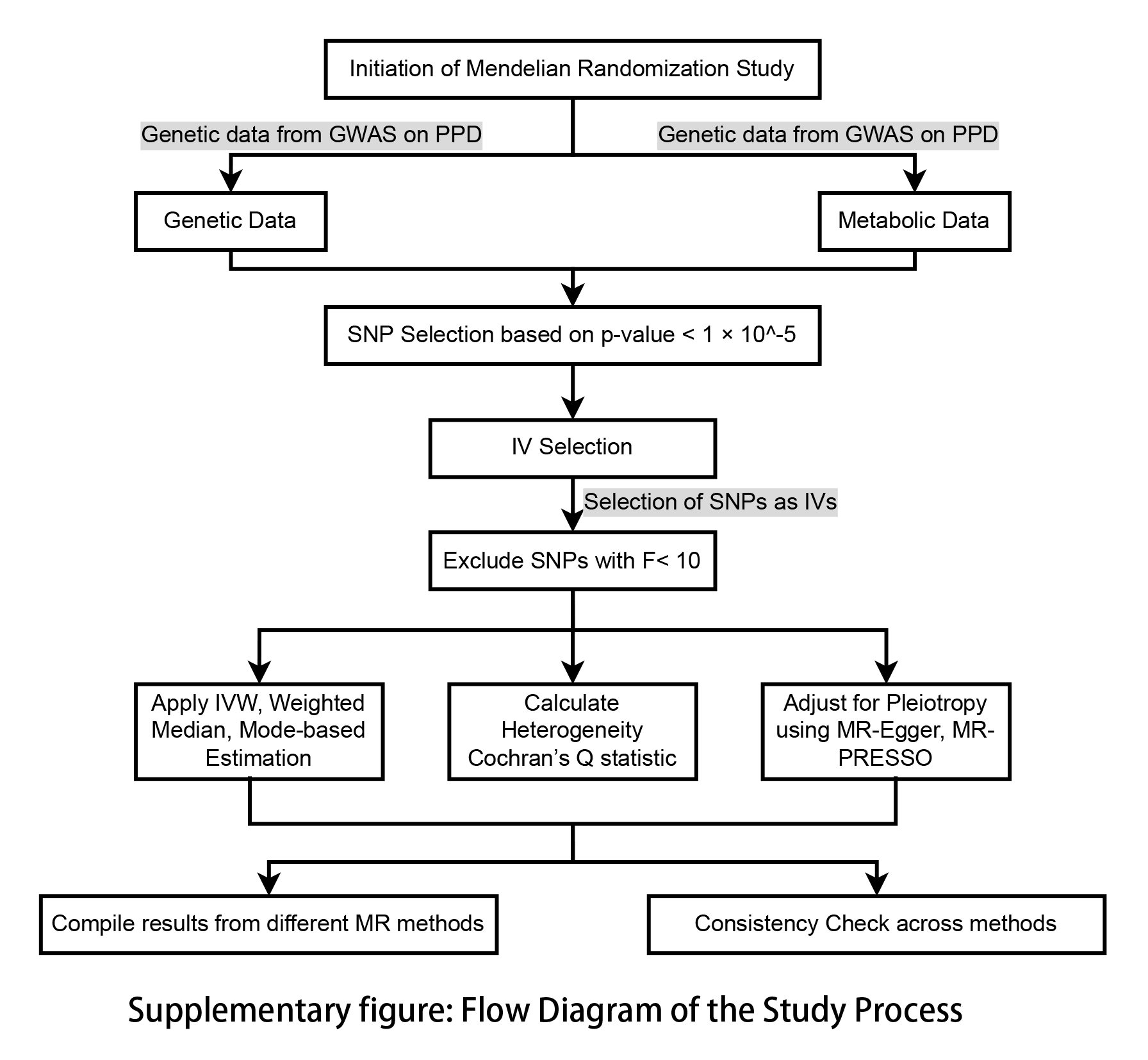

Supplement: Supplementary file 1 — Supplementary Material 1 [file 12884_2024_6628_MOESM1_ESM.png]
