# Supplementary material for: Blood metabolomic and postpartum depression: a mendelian randomization study
Source: BMC Pregnancy Childbirth. 2024 Jun 14;24:429. doi: 10.1186/s12884-024-06628-3 (PMC11177545; doi:10.1186/s12884-024-06628-3)
Supplement: Supplementary file 2 — Supplementary Material 2 [file 12884_2024_6628_MOESM2_ESM.docx]

**STROBE-MR checklist of recommended items to address in reports of Mendelian randomization studies**^1^ ^2^

| **Item No.** | **Section** | **Checklist item** | **Page No.** | **Relevant text from manuscript** |
| --- | --- | --- | --- | --- |
| 1 | **TITLE and ABSTRACT** | Indicate Mendelian randomization (MR) as the study’s design in the title and/or the abstract if that is a main purpose of the study | 1-2 | Blood Metabolomic and Postpartum Depression: A Mendelian Randomization Study  **Abstract**  **Background**  Postpartum depression is a complex mental health condition that often occurs after childbirth and is characterized by persistent sadness, anxiety, and fatigue. Recent research suggests a metabolic component to the disorder. This study aims to investigate the causal relationship between blood metabolites and postpartum depression using mendelian randomization (MR).  **Methods**  This study used a bi-directional MR framework to investigate the causal relationship between 1,400 metabolic biomarkers and postpartum depression. We used two specific genome-wide association studies datasets: one with single nucleotide polymorphisms data from mothers diagnosed with postpartum depression and another with blood metabolite data, both of which focused on people of European ancestry. Genetic variants were chosen as instrumental variables from both datasets using strict criteria to improve the robustness of the MR analysis. The combination of these datasets enabled a thorough examination of genetic influences on metabolic profiles associated with postpartum depression. Statistical analyses were conducted using techniques such as inverse variance weighting, weighted median, and model-based estimation, which enabled rigorous causal inference from the observed associations. postpartum depression was defined using endpoint definitions approved by the FinnGen study’s clinical expert groups, which included leading experts in their respective medical fields  **Results**  The MR analysis identified seven metabolites that could be linked to postpartum depression. Out of these, one metabolite was found to be protective, while six were associated with an increased risk of developing the condition. The results were consistent across multiple MR methods, indicating a significant correlation.  **Conclusions**  This study emphasizes the potential of metabolomics for understanding postpartum depression. The discovery of specific metabolites associated with the condition sheds new insights on its pathophysiology and opens up possibilities for future research into targeted treatment strategies. |
|  | **INTRODUCTION** |  |  |  |
| 2 | **Background** | Explain the scientific background and rationale for the reported study. What is the exposure? Is a potential causal relationship between exposure and outcome plausible? Justify why MR is a helpful method to address the study question | 3 | Postpartum depression is a serious mental health condition that affects new mothers. It is characterized by persistent feelings of sadness, anxiety, and fatigue, making it difficult for the affected women to perform daily care activities for themselves or their newborns. Other symptoms may include altered sleeping and eating habits, extreme irritability, and feeling of worthlessness or guilt. This condition has an impact not only on the mother's health but also on the baby's bonding and development. Early detection and treatment are critical to improve outcomes for both mother and child. The cause of postpartum depression is not fully understood, but recent research has begun to look into the role of metabolic changes in its development. Specifically, changes in blood metabolites have been linked to the onset and progression of postpartum depression, implying a potential metabolic component to the disorder. However, the causality of this relationship remains uncertain.  Blood metabolomics, which involves a thorough examination of metabolites in biological systems, has emerged as a valuable methodology for understanding the complex interplay between genetic factors, environmental influences, and disease processes. This analytical approach, which examines metabolite configurations, provides important insights into the metabolic anomalies associated with postpartum depression. Nonetheless, the field’s reliance on observational studies frequently presents difficulties due to confounding factors and issues of reverse causation.  The use of mendelian randomization (MR) in this context provides a distinct methodological advantage. MR uses genetic variants as instrumental variables (IVs), providing a novel way to decipher causal relationships between various exposures and outcomes while avoiding the inherent confounders and biases found in observational studies. |
| 3 | **Objectives** | State specific objectives clearly, including pre-specified causal hypotheses (if any). State that MR is a method that, under specific assumptions, intends to estimate causal effects | 3 | Our study uses the MR paradigm to investigate the potential causal relationship between metabolomic profiles and postpartum depression. Consequently, this study stands out as a pioneering effort in this emerging research arena, with the potential to significantly enrich our understanding of postpartum depression and its broader implications. |
|  | **METHODS** |  |  |  |
| 4 | **Study design and data sources** | Present key elements of the study design early in the article. Consider including a table listing sources of data for all phases of the study. For each data source contributing to the analysis, describe the following: |  |  |
|  | a) | Setting: Describe the study design and the underlying population, if possible. Describe the setting, locations, and relevant dates, including periods of recruitment, exposure, follow-up, and data collection, when available. | 4 | Data Sources for Postpartum Depression through Genome-Wide Association Studies (GWAS)  Summary statistics pertinent to postpartum depression were obtained from the GWAS database (https://gwas.mrcieu.ac.uk/). For postpartum depression, the sample consisted of 67,205 mothers with complete postpartum follow-up records, including 7,604 cases and 59,601 controls, totaling approximately 16,376,275 single nucleotide polymorphisms (SNPs). The study’s population was composed of people of European descent. Postpartum depression was defined using endpoint definitions approved by the FinnGen study's clinical expert groups, which included leading experts in their respective medical fields^[17]^. The FinnGen study established a strong framework for defining medical conditions, ensuring consistency and reliability in our diagnostic criteria.  Sources of GWAS Data for 1,091 Blood Metabolites and 309 Metabolite Ratios  The GWAS database, available at https://gwas.mrcieu.ac.uk/, served as a repository for summary statistics across a wide range of conditions. The GWAS summary datasets for 1,400 metabolites were extracted from the seminal study conducted by Chen et al, which represented the most extensive exploration to date of genetic influences on human serum metabolism. The exhaustive list of these 1,400 metabolites is provided in Supplementary Table S1. The demographic focus of this study was on people with European ancestry. |
|  | b) | Participants: Give the eligibility criteria, and the sources and methods of selection of participants. Report the sample size, and whether any power or sample size calculations were carried out prior to the main analysis | 4 | Data Sources for Postpartum Depression through Genome-Wide Association Studies (GWAS)  Summary statistics pertinent to postpartum depression were obtained from the GWAS database (https://gwas.mrcieu.ac.uk/). For postpartum depression, the sample consisted of 67,205 mothers with complete postpartum follow-up records, including 7,604 cases and 59,601 controls, totaling approximately 16,376,275 single nucleotide polymorphisms (SNPs). The study’s population was composed of people of European descent. Postpartum depression was defined using endpoint definitions approved by the FinnGen study's clinical expert groups, which included leading experts in their respective medical fields^[17]^. The FinnGen study established a strong framework for defining medical conditions, ensuring consistency and reliability in our diagnostic criteria.  Sources of GWAS Data for 1,091 Blood Metabolites and 309 Metabolite Ratios  The GWAS database, available at https://gwas.mrcieu.ac.uk/, served as a repository for summary statistics across a wide range of conditions. The GWAS summary datasets for 1,400 metabolites were extracted from the seminal study conducted by Chen et al, which represented the most extensive exploration to date of genetic influences on human serum metabolism. The exhaustive list of these 1,400 metabolites is provided in Supplementary Table S1. The demographic focus of this study was on people with European ancestry. |
|  | c) | Describe measurement, quality control and selection of genetic variants | 4-5 | Consistent with the current scientific literature, we set a significance threshold of IVs related to each trait at 1 × 10^− 5^. This process was made easier by using the R package “TwoSampleMR”, which helped refine the selection of SNPs.   1. Clumping for Linkage Disequilibrium   To address linkage disequilibrium (LD) between SNPs, we set a threshold of r^2 at 0.001 and specified a clumping proximity of 10,000 kb. LD measures the non-random association of alleles at different loci in a population, with an r^2 value of 1 indicating complete LD and a value of 0 indicating no LD.   1. Assessment of Instrument Strength   To mitigate bias stemming from weak instruments, we calculated the $R^{2}$ and F statistics for each SNP. The $R^{2}$ statistic quantifies the proportion of exposure variance explained by the IV, providing insight into the strength of the instrument. Conversely, the $F$ statistic evaluates the overall instrument strength, incorporating both the $R^{2}$ value and the sample size of the exposure group. SNPs with an F-statistic below 10 were excluded from subsequent analyses to minimize the risk of weak instrument bias, which can lead to inflated type I error rates and unreliable estimates.To calculate $R^{2}$ and $F$ statistics, follow the steps below:  $R^{2}=\frac{2\beta_{exposure}^{2}eaf_{exposure}\left( 1-eaf_{exposure} \right)}{2\beta_{exposure}^{2}eaf_{exposure}\left( 1-eaf_{exposure} \right)+2se_{exposure}^{2}samplesize_{exposure}eaf_{exposure}\left( 1-eaf_{exposure} \right)}$  $F=\frac{R^{2}\left( samplesize_{exposure}-2 \right)}{1-R^{2}}$  In these formulae:  • *β*_exposure_ represents the beta coefficient of exposure,  • *eaf_exposure_* is the effect of allele frequency of exposure.  • *se_exposure_* represents the standard error of exposure.  • *samplesize_exposure_* indicates the sample size of the exposure group.  We excluded SNPs with an F-statistic of less than 10 from our analysis to avoid weak instrument bias, which can inflate type I error rates and produce unreliable estimates. |
|  | d) | For each exposure, outcome, and other relevant variables, describe methods of assessment and diagnostic criteria for diseases | 4 | In this study, we used a bi-directional two-sample MR approach to investigate the hypothesized causal relationship between 1,091 blood-derived metabolites, 309 metabolite ratios, and postpartum depression.  The FinnGen study established a strong framework for defining medical conditions, ensuring consistency and reliability in our diagnostic criteria.  The demographic focus of this study was on people with European ancestry. |
|  | e) | Provide details of ethics committee approval and participant informed consent, if relevant |  | No relevant |
| 5 | **Assumptions** | Explicitly state the three core IV assumptions for the main analysis (relevance, independence and exclusion restriction) as well assumptions for any additional or sensitivity analysis | 4-5 | 1. There is a direct relationship between genetic variation and exposure.  2. There is no link between these genetic variants and confounders that could influence the exposure-outcome interaction.  3. Exclusiveness in the impact of genetic variation-driven exposure on outcome.  Cochran's Q statistic was used to investigate heterogeneity among the IVs. In cases of significant heterogeneity, the random-effects IVW model replaced the fixed-effects model. The MR-Egger method was used to address potential horizontal pleiotropy, with the intercept serving as an indicator of its existence. Furthermore, the MR-PRESSO approach was used to refine the analysis by identifying and eliminating outliers that could be attributed to pleiotropy. Funnel plots were used to ensure the consistency and reliability of the findings. |
| 6 | **Statistical methods: main analysis** | Describe statistical methods and statistics used |  |  |
|  | a) | Describe how quantitative variables were handled in the analyses (i.e., scale, units, model) | 5 | Analyses were conducted using the R programming environment (version 4.3.1). The investigation into the causal relationship between 1400 metabolites and postpartum depression was executed using methodologies such as inverse variance weighting (IVW), weighted median, and mode-based techniques, primarily through the TwoSampleMR package. |
|  | b) | Describe how genetic variants were handled in the analyses and, if applicable, how their weights were selected | 4-5 | Data Sources for Genetic Variants: Genetic variants were sourced from genome-wide association studies (GWAS). Specifically, summary statistics for postpartum depression and blood metabolites were obtained from the GWAS database.  Selection of Instrumental Variables (IVs): Genetic variants used as IVs were selected based on their association with the traits under investigation. A significance threshold for IVs was set at 1×10−51×10−5.  Clumping for Linkage Disequilibrium (LD): To address linkage disequilibrium between SNPs, a threshold of r2=0.001r2=0.001 was set, with a clumping proximity of 10,000 kb to ensure that the chosen SNPs are independent of each other.  Instrument Strength Assessment: The strength of the instruments was assessed by calculating the R2R2 and F-statistics for each SNP. The R2R2 statistic quantifies the proportion of exposure variance explained by the IV, providing insight into the strength of the instrument. SNPs with an F-statistic below 10 were excluded from subsequent analyses to minimize weak instrument bias. |
|  | c) | Describe the MR estimator (e.g. two-stage least squares, Wald ratio) and related statistics. Detail the included covariates and, in case of two-sample MR, whether the same covariate set was used for adjustment in the two samples | 4-5 | MR Estimator and Related Statistics:  MR Estimators Used: The analysis utilized several MR estimators, including inverse variance weighting (IVW), weighted median, and mode-based estimation methods. These methods are implemented primarily using the TwoSampleMR package.  Statistical Techniques: The document mentions the use of Cochran's Q statistic to assess heterogeneity among instrumental variables (IVs). In cases where significant heterogeneity was detected, a random-effects IVW model was used instead of a fixed-effects model. The MR-Egger method was also utilized to address potential horizontal pleiotropy, with the intercept serving as an indicator of its presence. Additionally, the MR-PRESSO approach was used to identify and adjust for outliers that could be attributed to pleiotropy.  Covariates in Two-Sample MR:  Handling of Covariates: The document does not explicitly detail the covariates included in the analysis or whether the same set of covariates was used for adjustment in both samples of the two-sample MR study. Typically, in two-sample MR analyses, it is crucial that both samples use a consistent set of covariates to prevent bias in the causal estimates. However, specific details on the covariates might need to be gleaned from further sections of the document or the original data sources used in the GWAS datasets. |
|  | d) | Explain how missing data were addressed | 4 | Complete Case Analysis: Analyzing only those cases where data are complete for all variables of interest. |
|  | e) | If applicable, indicate how multiple testing was addressed | 5 | Analyses were conducted using the R programming environment (version 4.3.1). The investigation into the causal relationship between 1,400 metabolites and postpartum depression was executed using methodologies such as inverse variance weighting (IVW), weighted median, and mode-based techniques, primarily through the TwoSampleMR package. |
| 7 | **Assessment of assumptions** | Describe any methods or prior knowledge used to assess the assumptions or justify their validity | 4-5 | Instrumental Variable Assumptions:  Relevance: Genetic variants (SNPs) were chosen as instrumental variables based on their strong association with the exposure (metabolites), ensuring that the relevance condition is met. The strength of these associations was verified using significance thresholds and the proportion of variance in the exposure explained by the IVs (denoted by R2R2 statistics).  Independence: The independence assumption was addressed by selecting SNPs that are not linked with any known confounders. This was facilitated by using clumping procedures to ensure that the genetic variants are independent not only of each other but also of external confounding variables. Linkage disequilibrium (LD) clumping was performed with an r2r2 threshold set to ensure minimal overlap in genetic architecture.  Exclusion Restriction: To justify the exclusion restriction, the study likely relied on biological knowledge indicating that the genetic variants affect the outcome only through their impact on the exposure. The absence of pleiotropic effects was statistically tested using the MR-Egger regression method, where the intercept can indicate the presence of directional pleiotropy if it significantly deviates from zero.  Sensitivity Analyses:  MR-Egger: This method provides a test for the presence of horizontal pleiotropy and can also give an estimate of the causal effect that is robust to certain violations of the exclusion restriction assumption.  MR-PRESSO: This test was used to detect and correct for outliers in the data, which could be indicative of pleiotropic effects or other violations of MR assumptions.  Cochran’s Q and I² Statistics: These were employed to assess heterogeneity among the genetic instruments, which can be an indicator of violations of the IV assumptions, especially the exclusion restriction. |
| 8 | **Sensitivity analyses and additional analyses** | Describe any sensitivity analyses or additional analyses performed (e.g. comparison of effect estimates from different approaches, independent replication, bias analytic techniques, validation of instruments, simulations) | 5 | Sensitivity Analyses and Additional Analyses:  MR-Egger Regression: This method was specifically employed to address potential horizontal pleiotropy. The MR-Egger intercept provides a test for the presence of pleiotropic effects that might bias the MR estimates. If the intercept significantly deviates from zero, it suggests the presence of directional pleiotropy, which might bias the causal estimates.  MR-PRESSO (Mendelian Randomization Pleiotropy RESidual Sum and Outlier): This tool was used to detect and correct for outliers in the instrumental variable analysis. Outliers can indicate the presence of pleiotropic effects or other issues with the genetic variants used as instruments. By identifying and adjusting for outliers, MR-PRESSO helps ensure that the analysis results are not unduly influenced by invalid instruments.  Weighted Median Estimator: This estimator provides a robust causal estimate that remains valid even if up to 50% of the information comes from invalid instruments. It was used as a complementary approach to the standard IVW method to confirm the findings under less stringent assumptions about the instrumental variables.  Cochran’s Q and I² Statistics for Heterogeneity: These statistics were calculated to assess the heterogeneity among the genetic instruments. Significant heterogeneity can indicate problems such as weak instruments or violations of the exclusion restriction assumption. The use of a random-effects model in the presence of significant heterogeneity helps to account for this and provides more reliable estimates.  Leave-One-Out Analysis: This approach was likely used to test the influence of individual SNPs on the overall MR results. By sequentially excluding one SNP at a time and recalculating the MR estimates, this analysis can identify if any single SNP disproportionately affects the results, which could indicate issues with specific instruments.  Comparison of Effect Estimates from Different MR Methods: The study compared the results obtained from different MR methods (IVW, MR-Egger, Weighted Median, and MR-PRESSO) to ensure consistency across different statistical approaches. Consistency in the findings across these methods adds confidence to the causal interpretations. |
| 9 | **Software and pre-registration** |  |  |  |
|  | a) | Name statistical software and package(s), including version and settings used | 5 | Statistical Software and Packages:  Software: R programming environment  Version: Version 4.3.1  Package: TwoSampleMR package |
|  | b) | State whether the study protocol and details were pre-registered (as well as when and where) |  | No relevant |
|  | **RESULTS** |  |  |  |
| 10 | **Descriptive data** |  |  |  |
|  | a) | Report the numbers of individuals at each stage of included studies and reasons for exclusion. Consider use of a flow diagram | 4  Supplemental image | The study’s population was composed of people of European descent. Postpartum depression was defined using endpoint definitions approved by the FinnGen study's clinical expert groups, which included leading experts in their respective medical fields. The FinnGen study established a strong framework for defining medical conditions, ensuring consistency and reliability in our diagnostic criteria  All in a flow diagram( Supplemental image). |
|  | b) | Report summary statistics for phenotypic exposure(s), outcome(s), and other relevant variables (e.g. means, SDs, proportions) | Supplemental Table S1-S6 | The full results and detailed data of the MR analysis are contained in Supplemental Table S3. |
|  | c) | If the data sources include meta-analyses of previous studies, provide the assessments of heterogeneity across these studies |  | No relevant |
|  | d) | For two-sample MR:  i.  Provide justification of the similarity of the genetic variant-exposure associations between the exposure and outcome samples  ii.  Provide information on the number of individuals who overlap between the exposure and outcome studies | 4-5 | Selection of Genetic Variants Based on Relevance  Assessment of Instrument Strength  To mitigate bias stemming from weak instruments, we calculated the $R^{2}$ and F statistics for each SNP. The $R^{2}$ statistic quantifies the proportion of exposure variance explained by the IV, providing insight into the strength of the instrument. Conversely, the $F$ statistic evaluates the overall instrument strength, incorporating both the $R^{2}$ value and the sample size of the exposure group. |
| 11 | **Main results** |  |  |  |
|  | a) | Report the associations between genetic variant and exposure, and between genetic variant and outcome, preferably on an interpretable scale | 6 | The IVW analysis for these metabolites yielded an aggregated score of:  2-o-methylascorbic acid levels (OR 1.10, p = 0.003188963),  N-formylmethionine levels (OR 1.17, p = 0.003186648),  2-hydroxyphenylacetate levels (OR 1.12, p = 0.003777128),  Inosine levels (OR 1.14, p = 0.002786266),  Inosine to EDTA ratio (OR 1.14, p = 0.001912067),  Carnitine to acetylcarnitine (C2) ratio (OR 1.26, p = 0.001447855).  Serine to alpha-ketobutyrate ratio (OR 0.78, p = 6.20E-05). |
|  | b) | Report MR estimates of the relationship between exposure and outcome, and the measures of uncertainty from the MR analysis, on an interpretable scale, such as odds ratio or relative risk per SD difference | Supplemental Table S1-S6 | The full results and detailed data of the MR analysis are contained in Supplemental Table S3. |
|  | c) | If relevant, consider translating estimates of relative risk into absolute risk for a meaningful time period |  | No relevant |
|  | d) | Consider plots to visualize results (e.g. forest plot, scatterplot of associations between genetic variants and outcome versus between genetic variants and exposure) | 7 | Figure 1: Forest plots showed the causal associations between metabolites on postpartum depression. |
| 12 | **Assessment of assumptions** |  |  |  |
|  | a) | Report the assessment of the validity of the assumptions | 6 | The Cochran’s IVW Q test, as detailed in Supplemental Table S4, revealed no significant heterogeneity in the IVs (p > 0.05). Furthermore, the MR-Egger regression intercept analysis, as shown in Supplemental Table S5, found no significant directional horizontal pleiotropy (p > 0.05). Furthermore, the MR-PRESSO global test (results in Supplemental Table S6) found no significant outliers, indicating a negligible presence of horizontal pleiotropy in the relationship between metabolites and postpartum depression (p > 0.05). |
|  | b) | Report any additional statistics (e.g., assessments of heterogeneity across genetic variants, such as *I^2^*, Q statistic or E-value) | 6  Supplemental Table S1-S6 | The Cochran’s IVW Q test, as detailed in Supplemental Table S4, revealed no significant heterogeneity in the IVs (p > 0.05). Furthermore, the MR-Egger regression intercept analysis, as shown in Supplemental Table S5, found no significant directional horizontal pleiotropy (p > 0.05). Furthermore, the MR-PRESSO global test (results in Supplemental Table S6) found no significant outliers, indicating a negligible presence of horizontal pleiotropy in the relationship between metabolites and postpartum depression (p > 0.05). |
| 13 | **Sensitivity analyses and additional analyses** |  |  |  |
|  | a) | Report any sensitivity analyses to assess the robustness of the main results to violations of the assumptions | 6  Supplemental Table S1-S6 | The Cochran’s IVW Q test, as detailed in Supplemental Table S4, revealed no significant heterogeneity in the IVs (p > 0.05). Furthermore, the MR-Egger regression intercept analysis, as shown in Supplemental Table S5, found no significant directional horizontal pleiotropy (p > 0.05). Furthermore, the MR-PRESSO global test (results in Supplemental Table S6) found no significant outliers, indicating a negligible presence of horizontal pleiotropy in the relationship between metabolites and postpartum depression (p > 0.05). |
|  | b) | Report results from other sensitivity analyses or additional analyses | 6  Supplemental Table S1-S6 | The Cochran’s IVW Q test, as detailed in Supplemental Table S4, revealed no significant heterogeneity in the IVs (p > 0.05). Furthermore, the MR-Egger regression intercept analysis, as shown in Supplemental Table S5, found no significant directional horizontal pleiotropy (p > 0.05). Furthermore, the MR-PRESSO global test (results in Supplemental Table S6) found no significant outliers, indicating a negligible presence of horizontal pleiotropy in the relationship between metabolites and postpartum depression (p > 0.05). |
|  | c) | Report any assessment of direction of causal relationship (e.g., bidirectional MR) |  | No relevant |
|  | d) | When relevant, report and compare with estimates from non-MR analyses |  | No relevant |
|  | e) | Consider additional plots to visualize results (e.g., leave-one-out analyses) | 8-9 | Figure 2: Scatter plots for the causal association between metabolites and postpartum depression  Figure 3: Leave-one-out plots for the causal association between metabolites and postpartum depression. |
|  | **DISCUSSION** |  |  |  |
| 14 | **Key results** | Summarize key results with reference to study objectives | 9 | In this study, we used a comprehensive approach that included GWAS data to investigate the causal relationships of 1,091 blood metabolites and 309 metabolite ratios with postpartum depression using a strong MR framework. Our study identified specific metabolites that were linked to an increased risk of postpartum depression. Notably, the ratio of Serine to alpha-ketobutyrate was found to be inversely correlated with the risk of postpartum depression. Conversely, a genetic predisposition to higher levels of 2-o-methylascorbic acid, N-formylmethionine, 2-hydroxyphenylacetate, and Inosine, as well as the ratios of Inosine to EDTA and Carnitine to acetylcarnitine (C2), has been linked to an increased risk of postpartum depression. These novel findings contribute significantly to a better understanding of postpartum depression’s pathophysiology, emphasizing the importance of metabolic pathways in disease risk. |
| 15 | **Limitations** | Discuss limitations of the study, taking into account the validity of the IV assumptions, other sources of potential bias, and imprecision. Discuss both direction and magnitude of any potential bias and any efforts to address them | 10 | However, our study does have limitations. Firstly, due to the small number and diversity of samples, our findings may require validation in a larger population. Furthermore, while the MR approach can reduce confounding and reverse causation, it still requires strong genetic IVs. Therefore, future studies should include larger sample sizes and more genetic variants to improve the reliability and universality of our findings. |
| 16 | **Interpretation** |  |  |  |
|  | a) | Meaning: Give a cautious overall interpretation of results in the context of their limitations and in comparison with other studies | 10 | However, our study does have limitations. Firstly, due to the small number and diversity of samples, our findings may require validation in a larger population. Furthermore, while the MR approach can reduce confounding and reverse causation, it still requires strong genetic IVs. Therefore, future studies should include larger sample sizes and more genetic variants to improve the reliability and universality of our findings. |
|  | b) | Mechanism: Discuss underlying biological mechanisms that could drive a potential causal relationship between the investigated exposure and the outcome, and whether the gene-environment equivalence assumption is reasonable. Use causal language carefully, clarifying that IV estimates may provide causal effects only under certain assumptions | 10 | Similarly, while the Serine to alpha-ketobutyrate ratio is not directly linked to postpartum depression in the available literature, Serine metabolism is recognized for its involvement in several psychiatric disorders, suggesting an avenue for further research into its specific role in postpartum depression[37-40]. Gestational diabetes, for example, can alter Serine and other amino acid levels, implying that metabolic diseases during pregnancy may predispose women to postpartum depression by disrupting key metabolic pathways involved in mood regulation. Conversely, our searches yielded no direct evidence linking metabolites such as 2-o-methylascorbic acid, N-formylmethionine, 2-hydroxyphenylacetate, the Inosine to EDTA ratio, or the Carnitine to acetylcarnitine (C2) ratio with postpartum depression. This does not rule out their involvement, but it does highlight the need for more research into the potential links between these metabolites and postpartum depression. The lack of direct associations in the literature suggests that these metabolites' roles in Postpartum depression are unknown, and additional research could greatly contribute to our understanding of postpartum depression's pathophysiology. This could include looking into how these metabolites interact with other metabolic pathways, as well as their potential role in the onset or progression of postpartum depression. |
|  | c) | Clinical relevance: Discuss whether the results have clinical or public policy relevance, and to what extent they inform effect sizes of possible interventions |  | No relevant |
| 17 | **Generalizability** | Discuss the generalizability of the study results (a) to other populations, (b) across other exposure periods/timings, and (c) across other levels of exposure |  |  |
|  | **OTHER INFORMATION** |  |  |  |
| 18 | **Funding** | Describe sources of funding and the role of funders in the present study and, if applicable, sources of funding for the databases and original study or studies on which the present study is based | 12 | This work was supported by the Medical Health Science and Technology Project Zhejiang Provincial Health Commission (Grant No. 2024KY454, 2023KY338, 2023KY337), Zhejiang Traditional Chinese Medicine Administration (2024ZL1058), and Zhejiang Provincial Basic Public Welfare Research Program (LQ21H160040). There are no conflicts of interest to declare. |
| 19 | **Data and data sharing** | Provide the data used to perform all analyses or report where and how the data can be accessed, and reference these sources in the article. Provide the statistical code needed to reproduce the results in the article, or report whether the code is publicly accessible and if so, where | 11  Supplemental Table S1  Supplemental R code with original results and images | Availability of data and materials  The datasets analyzed during the current study are available in the GWAS repository. The specific datasets used are:  Postpartum depression: finn-b-O15_POSTPART_DEPR  Blood Metabolomic: Supplemental Table S1  R code: Supplemental R code with original results and images |
| 20 | **Conflicts of Interest** | All authors should declare all potential conflicts of interest | 11-12 | Competing interests  The authors declare that they have no competing interests |

This checklist is copyrighted by the Equator Network under the Creative Commons Attribution 3.0 Unported (CC BY 3.0) license.

1. Skrivankova VW, Richmond RC, Woolf BAR, Yarmolinsky J, Davies NM, Swanson SA, et al. Strengthening the Reporting of Observational Studies in Epidemiology using Mendelian Randomization (STROBE-MR) Statement. JAMA. 2021;under review.

2. Skrivankova VW, Richmond RC, Woolf BAR, Davies NM, Swanson SA, VanderWeele TJ, et al. Strengthening the Reporting of Observational Studies in Epidemiology using Mendelian Randomisation (STROBE-MR): Explanation and Elaboration. BMJ. 2021;375:n2233.
